# Practitioner Views on the Impacts, Challenges, and Barriers in Supporting Older Survivors of Sexual Violence

**DOI:** 10.1177/1077801217732348

**Published:** 2017-10-25

**Authors:** Hannah Bows

**Affiliations:** 1 Durham University, UK

**Keywords:** aging and older people, sexual violence/abuse, elder abuse, adult protection

## Abstract

Despite half a century of research on both sexual violence and elder abuse, the intersection between the two remains largely unexplored. Using theoretical lenses of feminist criminology and critical feminist gerontology, this article explores the intersection between age and sexual violence drawing on interviews with 23 practitioners supporting older survivors (aged 60 and over). They reported physical and emotional effects of sexual violence leading to limited lifestyles, disengagement from social networks, and reliance on pathogenic coping strategies. Provision of effective support was complicated by challenges associated with aging bodies and the social stigma associated with both sexual victimhood and older age. Additional challenges lay in supporting older male survivors and those living with dementia. The article ends by discussing implications for practice and an agenda for future research.

## Introduction

Sexual violence is recognized as a global public health problem affecting millions of people. Sexual violence, used interchangeably here with the term sexual abuse, is defined as

any sexual act, attempt to obtain a sexual act, unwanted sexual comments or advances, or acts to traffic, or otherwise directed, against a person’s sexuality using coercion, by any person regardless of their relationship to the victim, in any setting, including but not limited to home and work. (World Health Organization [WHO], n.d.-c, p. 149)

The WHO estimates that more than 35% of women worldwide will experience at least one instance of sexual violence during their lifetime (WHO, 2013). In England and Wales, official measures of sexual violence are drawn from official police records and the Crime Survey for England and Wales (CSEW). Between 2009 and 2012, on average, one in 40 women and one in 250 men had been the victim of a sexual offense or attempted sexual offense in the previous 12 months (Home Office, 2013b). This represents an average of 404,000 women and 72,000 men per year.

These figures from the CSEW and WHO include crimes not reported to the police and go some way to address the widely acknowledged chronic underreporting of sexual violence against women (García-Moreno, 2013) and men (Sleath & Bull, 2010). Recent figures from England and Wales suggest a reporting rate of only 15% among women experiencing the most serious sexual offenses (Home Office, 2013b). However, the CSEW excludes the experiences of those living in communal establishments including care homes, and limits data collection on domestic abuse and sexual violence to those aged 16-59. These limitations are both symptomatic of, and contribute to, the exclusion of older people from the field of legitimate public concern in the arena of sexual violence. Sexual violence against older people sits awkwardly between the more established fields of elder abuse and domestic violence, and represents a chasm between elder abuse and other forms of violence against women across the life span (Roberto, McPherson, & Brossoie, 2014; Walsh et al., 2007) rendering older women’s experiences invisible (Roberto et al., 2014). This has important implications for older survivors of sexual violence, and for practitioners providing support services.

Feminist theories have been at the forefront of sexual violence research and are relevant to sexual violence against older women due to their emphasis on both the individual and structural levels of multiple oppressions in a patriarchal society that may be experienced by women across the life course (Browne, 1998, Collins, 1986). However, feminist theories have been criticized for failing to consider how other characteristics and social factors contribute to the victimization and experiences of women (Crenshaw, 2003), including age (Jones & Powell, 2006). Consequently, a critical feminist gerontological perspective, which is “grounded in feminist theory and critical gerontology” and focuses on “power relations and intersecting oppressions across the life course” (Hooyman, Browne, Ray, & Richardson, 2002, p. 3), is needed. An analytical framework which incorporates gender and age is needed to fully examine and understand sexual violence against older people.

Following a brief review of literature to inform understanding of the challenges in addressing the intersection between older age and sexual violence, this article presents the findings of an empirical study designed to extend knowledge and understanding of sexual violence against older people before considering implications for policy, practice, and for future research.

## The Literature

Research on the effects of abuse and violence against older people has developed considerably over the last two decades (Anetzberger, 2005; Lindbloom, Brandt, Hough, & Meadows, 2007). In the area of sexual abuse, a number of studies have attempted to estimate the prevalence of sexual violence against older people (Bows & Westmarland, 2017; Cannell et al., 2014; Luoma et al., 2011; Naughton et al., 2012; O’Keeffe et al., 2007; Scriver, Mears, & Wallace, 2013), although a lack of consensus prevails (for a review, see Bows, 2017b; Fileborn, 2016). The existing research has found the types and nature of sexual violence experienced by older women are similar to that of younger women. For example, the vast majority of victims are female and perpetrators are male (Bows & Westmarland, 2017; for a review, see Bows, 2017b; Fileborn, 2016), rape tends to be the least commonly reported offense (Ball & Fowler, 2008), and the majority of victims know their perpetrator (Bows, 2017b; Fileborn, 2016; Mann, Horsley, Barrett, & Tinny, 2014). By contrast, there is still a poverty of research examining the effects of sexual violence on older people, their support needs and coping strategies, and experiences of accessing support services.

The small pool of existing literature indicates that older women who experience sexual violence are more prone to genital trauma than younger women (Bows, 2017b; J. S. Jones, Rossman, Diegel, Van Order, & Wynn, 2009; Morgan, Dill, & Welch, 2011; Muram, Miller, & Cutler, 1992; Ramin, 1997). A recent study by Soares et al. (2010) reported somatic symptoms (such as body aches, pains) were linked to sexual abuse. Morgan, Dill, and Welch (2011) report bruising as an impact observed at a higher rate in postmenopausal women. Jeary (2005) reports a range of long-term physical and mental health problems following older women’s experiences of sexual violence, which result in a range of impacts requiring painkillers as a result of injuries, or creating long-term problems such as suffering incontinence since the assault. Psychological consequences include flashbacks and nightmares, problems sleeping, anxieties and fears about leaving their home, or, if the attack happened in their home, fear of living in the property resulting in some older women moving house or into residential care settings (Jeary, 2005).

Little is known about older people’s support needs, or the effectiveness of responses by professional practitioners, following experiences of sexual violence (Bows, 2017b; Mann et al., 2014) or intimate partner violence (Brossoie & Roberto, 2016). Despite increased research examining elder abuse, there remains significant gaps in the literature specifically examining sexual violence against older people and consequently our understanding of these issues remains limited (Fileborn, 2016). Moreover, there is currently no existing literature on the experiences of practitioners in supporting older survivors of sexual violence, although previous research with younger survivors of domestic and sexual violence have found that some experience vicarious trauma or emotional distress (Baird & Jenkins, 2003; Dworkin, Sorell, & Allen, 2016; Schauben & Frazier, 1995; Slattery & Goodman, 2009).

Interpreting evidence and arguments about sexual violence against older people is complicated by the use of different terms reflecting different academic fields of study, different theoretical standpoints, and different areas of jurisdiction. Jones and Powell (2006), focusing on professional practice in health care, argue that the relevance of sexual violence in relation to aging and vulnerability has been overlooked both in policy and practice, and in mainstream criminology and feminism. Hollomotz (2009) shows how the term “sexual abuse” is commonly used to describe experiences of sexual violence against young people, people with disabilities, and older adults, whereas “harassment,” “assault,” and “rape” are used to describe the experiences of nondisabled adults (p. 101). The differences in terminology, she argues, reflect the ways in which disabled and older people are effectively denied adult social status that confers citizenship rights, an argument reinforced by the institutional ageism reflected in the CSEW.

This study aimed to address the current gaps in knowledge through qualitative interviews with practitioners working in sexual violence organizations to examine practitioner perceptions of the effects of sexual violence on people aged 60 and over, the impact that age has on experience and challenges this creates in accessing and providing support services, and the current gaps in service provision. This is the first published study to include a focus on the challenges that the age of survivors can create for practitioners working in sexual violence organizations. Focusing on the intersections of age and gender, this article responds to calls from previous researchers (Fileborn, 2016; Jones & Powell, 2006; Whittaker, 1995) to examine sexual violence against older people through a feminist lens.

## The Study

The study reported here adopts a theoretical framework informed by feminist criminology (Burgess-Proctor, 2006) and critical feminist gerontology (Ray & Fine, 1999), paying attention to the distinctive experiences of older women and older men. Garner (2014) highlights the similarities between gerontology and feminist theories. Common goals include the development of social consciousness about inequities, utilization of theories and methods that accurately depict life experiences, and promotion of change in conditions that negatively affect older people or women. This critical feminist gerontology perspective “sensitizes us to other power relations such as age relations and is appropriate for studying both female and male victims of sexual violence, even though women are the majority of victims” (Calastani, 2004 p. s305). Thus, by examining sexual violence against older people through a critical feminist gerontology lens, both gender and age become central. Filtered through the accounts of practitioners working directly with older survivors, the study also identifies challenges faced by those with roles in collecting forensic evidence and in providing support to older survivors.

Before proceeding it is important to explain that there are no single definitions of the terms “older,” elder abuse, sexual violence, or domestic violence. In this study, “older” refers to people aged 60 and over (WHO, n.d.-b). Elder abuse is understood as “a single or repeated act, or lack of appropriate action, occurring within any relationship where there is an expectation of trust which causes harm or distress to an older person” (WHO, n.d.-a). Domestic violence, used interchangeably with the terms “domestic abuse” and “intimate partner violence,” is used to refer to “any incident or pattern of incidents of controlling, coercive, or threatening behaviour, violence or abuse between those aged 16 or over who are, or have been, intimate partners or family members regardless of gender or sexuality” (HM Government, 2013).

Drawing on a larger study exploring gaps in knowledge about sexual violence against older people, this article focuses on interviews with 23 practitioners working directly with survivors of sexual violence. As Ullman and Townsend (2007) observe, practitioners working in organizations that serve victims and survivors of sexual violence can be a useful source of information in identifying barriers faced by victims in reporting assault to criminal justice agencies and in accessing support. They are important sources of information about the challenges service providers face when assisting survivors.

Ethical approval for the study was granted by the School of Applied Social Science (Durham University) Ethics Committee. The sample of practitioners was recruited using purposive and snowball sampling techniques through which those first approached identified further potential participants (Atkinson & Flint, 2001; Everitt & Howell, 2005). Using established feminist activist networks, initial contact was made with Rape Crisis, Sexual Assault Referral Centres (SARCs), and domestic violence organizations in England and Wales resulting in 19 practitioners agreeing to take part. A further four participants were recruited through the use of Twitter to promote the research. This proved particularly useful in making contact with practitioners outside established networks, such as those working with male survivors.

The final sample of 23 practitioners included 21 working across a range of roles, including counselors, therapists, helpline volunteers, clinical leads, and forensic medical examiners, and management roles, including volunteer coordinators and chief executive officers (CEOs), and two working independently as a trauma therapist and counselor, who practiced predominantly with male survivors (see Table 1). The latter two practitioners were male and the remaining 21 practitioners were all female. The age range of practitioners was between mid-20s and mid-60s, though the majority were aged between 35 and 55. To protect the identity of participants and the older people they had supported, sample members are described simply by the use of a participant letter and their broad job title and organization type.

**Table 1.** Practitioner Role and Organization Type.

Practitioner assigned letter	Practitioner role	Practitioner organization
A	ISVA and IDVA	Rape Crisis Organization
B	Clinical lead	Rape Crisis Organization
C	Independent trauma counselor	Sexual Violence
D	CEO	Rape Crisis Organization
E	Forensic medical examiner	SARC
F	Volunteer coordinator	Rape Crisis Organization
G	Counselor	Rape Crisis Organization
H	Helpline advisor	Rape Crisis Organization
I	Independent trauma counselor	Sexual Violence
J	Service manager	SARC
K	Volunteer coordinator	Rape Crisis Organization
L	Counselor	Rape Crisis Organization
M	Refuge manager	Domestic Violence
N	Support worker	Domestic Violence
O	Manager	SARC
P	CEO	Rape Crisis Organization
Q	Support worker	Domestic Violence
R	Manager	SARC
S	CEO	Rape Crisis Organization
T	Information and advice support	Domestic Violence
U	Director	Rape Crisis Organization
V	CEO	Rape Crisis Organization
W	GP	SARC

*Note*. ISVA = Independent Sexual Violence Adciser; IDVA= Independent Domenstic Violence Adviser; CEO = chief executive officer; GP = General Practitioner; SARC = Sexual Assault Referral Centre.

Five semistructured interviews with practitioners were conducted face-to-face and 18 by telephone where geographical distance made face-to-face encounters impractical. Prior to each interview, practitioners were provided with information sheets about the study and consent was obtained at the beginning of each interview.

The interviews, lasting between 30 and 90 min, explored (a) practitioners’ observations and understandings of the effects of sexual violence, and the coping strategies adopted by older people, and (b) challenges in providing support to older survivors. An interview schedule was used, which consisted of two sections: The first section asked general questions about the practitioner’s organization, role, length of service, and proportion/frequency they supported survivors over the age of 60 for sexual violence experienced in later life over the previous 5 years; the second section focused on the practitioner’s views of older survivors reporting/disclosing and accessing services, incorporating questions on the impacts or challenges practitioners had observed in relation to older survivors and their own experiences of supporting them.

Face-to-face interviews were audio-recorded and transcribed, whereas telephone interviews were typed verbatim, a process enabled by the author’s earlier training as a legal secretary. Transcripts were analyzed using a critical feminist framework; the subjective experiences of practitioners were central to the analysis which was concerned with developing rich descriptions of practitioner perspectives, views, and experiences, with particular focus on gender, age, and the effects of these intersections. Coding began by reading the transcripts line by line, attaching descriptive labels to each phrase, sentence, and later to larger sections of text, to identify and interpret the experiences and perspectives of the practitioners (Ritchie & Spencer, 2002). As this research formed part of a larger doctoral study (Bows, 2017a, 2017b; Bows & Westmarland, 2017), coding was done by an individual researcher. Themes were then drawn out of the transcripts based on these codes. For example, all of the practitioners described different physical challenges that could create difficulties or barriers for older people accessing support services (such as reduced mobility, hearing, or sight problems). These were all coded as physical challenges and grouped under this as a theme.

## Findings

The starkest finding was the poverty of practice experience in supporting older survivors of sexual violence, mirroring recent research examining intimate partner violence in later life (Brossoie & Roberto, 2016). Practitioners were not asked to provide specific numbers of older people accessing their services, but were asked at the beginning of the interview how much experience they had supporting older people who had experienced sexual violence in older age in the previous 5 years. In total, the 23 practitioners had seen approximately 100 older people in the previous 5-year period. Practitioners working in SARCs estimated they had encountered, at most, 10 older people in the course of the previous 5 years, whereas those working in rape crisis centers, domestic violence refuges, and independently had worked with between one and five older people in the previous 5-year period. This is unsurprising given the lack of attention to sexual violence against older people in crime statistics and social care policies and procedures. But it also presents an opportunity to begin to develop insights into the worlds of those who attempt to support older people experiencing sexual violence, how they interpret older people’s experiences, and the challenges they encounter in engaging with older survivors.

### Practitioners’ Insights Into the Effects of Sexual Violence on Older People

Practitioners described observing a range of overlapping physical and emotional effects of sexual violence with subsequent lifestyle-related effects for older survivors. Physical effects included bruising and cuts, genital injuries, and broken bones, creating long-term health conditions including incontinence, and exacerbating existing injuries or conditions such as arthritis. Some practitioners had observed significant declines in survivors’ health while engaging with the support service, which they attributed at least partly to their experience of sexual violence. As one practitioner explained,

One woman experienced her first sexual violence in 2013, having had nothing prior to this, and at the time she was 71. By the June of 2014 she was diagnosed with depression and PTSD, had 2 heart attacks in that time, and was nocturnally incontinent. (Participant A, ISVA/IDVA)

And a trauma counselor described his experience of counseling an older man who suffered from arthritis in his shoulder:

He was being raped and this guy kept moving his shoulder to make him scream, because he didn’t scream when being penetrated. So he made him scream by moving his shoulder. (Participant C, Trauma Counselor)

The severity of violence was also noted by other practitioners. Participant C also described the experience of an older woman raped by a younger acquaintance who broke both her hips during the rape.

One thing [which is] alarming about perpetrators who target older women is that the rapes are quite ritualistic and violent and sinister. (Participant B, Clinical Lead)

Emotional effects described by practitioners were similar to those observed in younger survivors, including posttraumatic stress disorder (PTSD), depression, anxiety, sleep disturbance, and stress (Ullman, 2010). All practitioners felt these symptoms were amplified for older people arguing that, because of their age, they had fewer opportunities to draw on, or develop, supportive networks, for example, through employment or social relationships.

Both the physical and emotional effects of sexual violence were thought to result in a number of lifestyle changes for older survivors leading to social isolation (Lachs & Pillemer, 2004). Practitioners referred to the development of agoraphobia, altered relationships with family or friends, and disengagement from support organizations. The following example of an older woman who was raped while on holiday abroad underlines the perception among practitioners that some impacts of sexual violence may have been shaped by age:

I think [the effects are] different to younger women . . . the woman I worked with will never go abroad again. For younger women it is easier to move on but older women will totally change their behavior. (Participant D, CEO Rape Crisis)

Practitioners’ understandings of coping strategies identified pathogenic strategies that masked rather than addressed the experiences of sexual violence. Although many of these strategies were similar to those adopted by younger survivors (Ullman et al., 2013)–for example, taking prescription medication for pain relief or depression–six practitioners had noted the use of alcohol as a coping mechanism by older survivors. For example:

They will use alcohol as a way of coping, which induces a lot of shame. They feel uncomfortable accepting they use this as a way to cope. (Participant B, Clinical Lead)

The use of such strategies by older survivors was perceived as introducing further challenges or exacerbating existing strategies established over time as a way of managing everyday stress or other traumatic experiences. Although these are not unique to older people, such coping mechanisms can exacerbate existing age-related health problems (e.g., cardiovascular disease).

### Challenges in Providing Support to Older Survivors

Three broad sets of challenges emerged in practitioners’ accounts of providing support to older survivors: physical, emotional, and social/cultural. A further specific challenge was raised in relation to working with older people living with dementia, and reference was made to conspicuous absence of older women from black and minority ethnic groups accessing sexual violence support services. The following sections are constructed to reflect the broad areas of challenges and to demonstrate the connections between them.

#### Physical challenges

Physical challenges in supporting older survivors were cited by all practitioners and centered around poor physical health and physical impairments, either preexisting or the result of the sexual violence.

I think they are more likely to have more health issues. It may be that we have to think about access and that sort of thing. It could be they are more hard of hearing, [or they] may need more support getting to the centre. The older you get the more difficult it is to access [support]. (Participant D, CEO Rape Crisis)

For those working in SARCs, physical impairments were experienced as presenting challenges both for survivors and practitioners. A forensic medical examiner explained,

To do a forensic examination, they need to climb on a couch, open [their] legs, etc. Just physically managing them in an environment is challenging. Arthritis or physical conditions can limit mobility, and so can emphysema. Again, they are limited with getting to the SARC so [I] might have to see them in their own home which isn’t forensically ideal. (Participant E, Forensic Medical Examiner)

Sight and hearing impairments were perceived as creating barriers to accessing support services, although none of practitioners felt inhibited in providing support once survivors had accessed the service. The premises of all the organizations represented by practitioners in the sample were wheelchair accessible and provided information in a range of accessible formats, for example, leaflets in different languages, including minority languages and in braille. The practitioners claimed that all their services were tailored to individual needs, and were keen to point out that many younger survivors also had access requirements or specific service needs. However, they were conscious that outreach support had been particularly useful with older survivors, where physical conditions or mental health challenges prevented them from attending agency premises.

#### Emotional challenges

Emotional challenges were described as mirroring those of younger survivors: a sense of shame, fear, anxiety, and self-blame (K. G. Weiss, 2010). However, practitioners felt that emotional challenges were magnified for some older survivors because the prevailing cultural norms in their earlier lives serve to inhibit disclosure or discussion of sexual violence (Fivush, 2010). In particular, practitioners felt sexual violence carried a lot of stigma for the victim:

The stigma that they are older. The shame. Again, it is a generational thing. They keep it from their children and having to go through that process. The whole process is horrific and for an older woman, having swabs and people coming and looking at your body. There is a different embarrassment attached to it when you are young. (Participant F, Volunteer Counselor)

Practitioners were unanimous in articulating the importance of ensuring that the support offered to survivors, and the way it is delivered, is sensitive to the stigma associated not only with sexual abuse but also with older age and nudity. For example, all of the clinical practitioners felt that older people may find physical examination more comfortable and less embarrassing by reducing the age difference between the individual survivor and the clinical practitioner.

#### The challenge of supporting individuals living with dementia

Practitioners expressed particular challenges in providing effective support for older survivors living with dementia. Seven practitioners had worked with older survivors with memory function issues, including dementia. A key concern was that they may not be believed when they disclosed abuse, their accounts dismissed by carers, family, or professional bodies.

[She was] very unwell with dementia and I think the trouble with that is that people tend to dismiss the presentation of something. I look at it that something must have happened that this person is bringing it up. Perhaps when they were able minded they couldn’t talk about it because of the stigma. We are not there to disprove something. (Participant G, Counselor)

In contrast, practitioners who had provided counseling support to individuals living with dementia referred to the inherent ethical dilemmas of working with people who struggle to remember the details of the sexual violence:

I have two more which are linked to dementia. They had been raped but couldn’t remember it, which I quickly brought to a halt because there was no value. Whenever we got anywhere near, with the chap having memory problems, it had no value. There are problems when you are dealing with Alzheimer’s or dementia that to constantly remind this guy that he had been raped was totally wrong and it outweighed any benefit there was of telling him. (Participant C, Independent Trauma Counselor)

Further challenges included the complexities of gaining consent to perform a forensic medical examination when the victim may not have the capacity to give their own consent:

From a consent perspective, how you manage a best interest decision can be challenging. To do an examination I should have informed consent. So, if they have dementia and don’t understand what I am doing and the implications, then you are having to do a best interests decision and then you are getting social services and those sort of people involved. (Participant E, Forensic Medical Examiner)

These words offer an interesting insight into the challenges of interprofessional working that are central to effective adult safeguarding (Abley, Bond, & Robinson, 2011). Not only does the forensic examiner refer to social services as “those sort of people” but also suggests that the role for social services may be limited to best interests decisions in situations where individuals lack mental capacity.

There was concern about a lack of clear guidance or training in relation to managing these challenges where practitioners felt the least confident in their knowledge of how best to support survivors and the most appropriate services to refer to.

#### Sociocultural challenges

Ageist beliefs and attitudes presented further barriers for older people in accessing support following sexual violence. The popular myth that rape is linked to sexual desire, together with perceptions of older people as sexually undesirable (Bows and Westmarland 2017) presented particular challenges:

Rape being a compliment—“nobody would rape her because she is so old.” People assuming older women won’t get raped for that reason because they aren’t sexually desirable. (Participant H, Helpline Volunteer)

These beliefs made it difficult for older survivors to understand their own experiences of sexual violence. Helping them to develop that understanding became a central role for counselors and support workers. As one explained,

I think they find it hard to conceptualize what has happened to them, because of how they look at things and how they look at themselves. We all get to an age where we think we are past it, not attractive, etc. So why would anyone want to engage in that? They see it firstly and foremost as a desire, not as something of control and manipulation. (Participant L, Counselor)

The two practitioners who worked with male survivors experienced particular challenges in this sense.

All 23 practitioners felt this lack of awareness, together with ageist attitudes depicting older people as vulnerable, frail, and undesirable created barriers to providing support. Some, particularly counselors, found it emotionally challenging themselves:

Personally [I] found it slightly harder, a hurdle I had to overcome as I have always looked up to people who are older than me so quite difficult providing support to someone older than me. (Participant H, Helpline volunteer)

A trauma counselor who worked with male survivors had found it hard to get past his own assumptions:

Over the years I have dealt with over 60s but that tends to be historic abuse so on that level I was OK, but I found it difficult to try and contextualize sometimes what had happened to those aged 60 or over. I have less experience supporting older people with recent abuse. I don’t think there has ever been any real awareness around it which means there is no text I can turn to, no research. I am usually pretty good at looking at research—it just doesn’t exist. So I felt out of my depth. I felt some kind of training should be there. (Participant I, Independent Trauma Counselor)

All of the practitioners felt that older people may be uncomfortable sharing highly sensitive and distressing experiences with younger counselors or support workers who may be a similar age to their children or grandchildren. As one practitioner explained,

The client I had who was in her 70s was raped and one of the things she said she liked was that I was older. She also went to quite a lot of groups and one of the things she said was that there seemed to be a lot of younger workers there and she personally found that harder, she had never disclosed to any of the workers because she felt they were too young. (Participant D, CEO Rape Crisis)

In addition to concerns about older people’s reluctance to share distressing experiences with those of a younger generation, the practitioners were conscious that they themselves may find it more difficult to appreciate the sociocultural contexts in which older survivors had grown up, where public discussion of sexual violence was subjected to what Romito (2008) has described as “a deafening silence.”

## Gaps in Service Provision

Beyond the challenges described thus far, practitioners in this study identified a number of weaknesses or gaps in support services. These included lack of awareness among older people of the existence of support services. Responsibility for this lack of awareness was seen as multifaceted, but all of the practitioners felt they, or their organizations, had a role to play in raising awareness.

The gap is us—women knowing where they can access emotional support. Maybe a generation thing, assuming they don’t want to talk about it or them not wanting to talk about it. (Participant J, Service Manager)

There was unanimity among practitioners that there was insufficient collaboration, locally and nationally, between age-related organizations such as Age UK, rape crisis centers, SARCs, and domestic violence organizations:

I think gaps are probably [the] lack of information into services that actually support older people—we are not targeting them enough and getting where we should. As an organization we should be going to Age UK so women know we are here, they can get support. Raise awareness; we are great at doing it for younger people but I think we need to make sure women who are older can access services. (Participant K, Volunteer Coordinator)

Although a number of practitioners referred generally to older women survivors, five commented in particular about the gap in services for minority groups of older survivors, including men and older people from black and minority ethnic (BME) groups.

A large chunk of my time has been spent with BME women and I don’t think I saw a single BME woman aged 60+ come forward. [It’s] different with BME because of cultural issues, at risk of further victimization if [they] were to report. (Participant B, Clinical Lead)

These practitioners’ accounts, based on limited experience in working with older survivors of sexual violence, convey powerful messages about the challenges that serve to reinforce the marginalization of sexual violence against older women and men. The following section addresses the implications of these messages for practitioners working in roles supporting sexual violence survivors.

## Discussion and Implications for Policy and Practice

By examining the impacts of sexual violence on older people and the challenges in accessing support through a critical feminist gerontology lens, it can be argued that older survivors can experience a double disadvantage or “double jeopardy” (Mann et al., 2014, p. 20) rooted in ageism and sexism. The lack of awareness and acceptance of sexual violence in later life reflect ageist and sexist attitudes toward older people. Both age and gender intersect to create specific impacts, challenges, and barriers. In terms of practitioner responses to older survivors, this can have specific consequences in practice delivery. As Goldblatt, Band-Winterstein, and Alon (2016) point out, “In intervention with young women, social empowerment is the central principle based on social ideology that maintains that women are confident, capable of taking responsibility for their own fate and making a change in their lives” (p. 14). However, ageist constructions of older people as weak and vulnerable based on a perception of aging as a process of decay, decline, and deterioration (Jones & Powell, 2006) may affect the type of intervention offered and/or the success of support services. Social attitudes and responses to those who experience sexual violence, still largely characterized by shame and silence (Patterson, 2016), are compounded by ageism that portrays older people as asexual and therefore outside the accepted boundaries of concern as survivors of sexual violence. Moreover, “real rape” stereotypes and ageist attitudes were unanimously referred to by practitioners as creating challenges for older survivors who do not fit the “real rape” model of a young, white, female victim who is attacked by a young male stranger, motivated by sexual desirability (Bows & Westmarland, 2017). Both police and media campaigns and coverage of cases tend to reinforce these stereotypes, which can have negative impacts on victims who may be less likely to be believed and/or be reluctant to report cases that are not in keeping with the stereotype (McMillan & Thomas, 2009). This has important implications for older people who may be reluctant to report or disclose sexual violence because they do not fit the “real rape” model.

Physical conditions and issues associated with aging may affect the interventions available to, or suitable for, older survivors. As the population rapidly ages, an increasing number of older people, in particular women, will be living longer and managing a range of physical and psychological health conditions. Such conditions, such as arthritis, heart problems, limited mobility, disability, and dementia can result in specific impacts following sexual violence, which create barriers and challenges in accessing support. Research has shown that disabled women (Khalifeh, Howard, Osborn, Moran, & Johnson, 2013; Macdowall et al., 2013; Smith, 2008) and men (Mitra, Mouradian, Fox, & Pratt, 2016) are more likely to experience domestic or sexual violence and may be less likely to seek help than nondisabled women . Furthermore, recent research examining elder abuse has drawn attention to issues of old age, which can add to the complexities of the abuse experienced: reduced functioning, illness, quality of life, mortality, and reduced feelings of hope (Band-Winterstein et al., 2014).

In the context of sexual violence, age may increase the chances of health complications such as genital trauma and long-term urinary or colorectal problems following rape or sexual assault, or may exacerbate existing physical or mental health problems such as heart conditions, arthritis, or dementia. This can create difficulties for survivors in accessing support, but can also create challenges for practitioners in providing support. Examples provided by practitioners included difficulties in conducting forensic medical examinations where the older person had physical conditions making it difficult to get onto medical beds and, where the older person had dementia, problems around gaining consent to perform examinations. For counselors, dementia was also a concern as there are ethical considerations in providing counseling support to someone who, for example, cannot always consistently remember they have been raped. Concerns about whether professionals are alert to the signs of physical and sexual abuse among older people suffering with dementia have already been raised (Flannery, 2003); however, there remains a lack of research and guidance in this area.

The lack of available training and guidance on these problems is symptomatic perhaps of the low numbers of older people who access support services for rape, mirroring intimate partner violence research (Brossoie & Roberto, 2016), but also arguably the wider sociocultural attitudes around older people and sexual violence. Consequently, the lack of awareness of sexual violence in later life may prevent older survivors from reporting or accessing support services, and according to practitioners in this study, may reinforce shame and embarrassment, creating further barriers. For older male victims, generational norms and attitudes around masculinity and homosexuality may create further barriers making disclosures difficult and increasing feelings of shame and embarrassment, that must be factored into the support provided by practitioners, particularly counselors. Practitioners felt that emotional challenges were magnified for some older survivors because the prevailing cultural norms in their earlier lives served to inhibit disclosure or discussion of sexual violence (Fivush, 2010). Moreover, the real rape stereotype may invalidate older survivors’ experiences and lead to further confusion and self-blame.

Further concerns were expressed by practitioners lacking guidance in supporting older survivors living with dementia in relation to possible misinterpretation of disclosures of sexual abuse and fear that attempts to support survivors with dementia may create unnecessary anxiety. In this embryonic area of practice, learning from safeguarding reviews and best practice knowledge needs to be disseminated and shared between social care, health, criminal justice, sexual violence and age-related agencies, and practitioners who may come into contact with older survivors of sexual violence. Manthorpe and Martineau’s (2016) analysis of serious case reviews involving people with dementia identified the need for social workers to develop their forensic skills of evidence collection, synthesis, and analysis as well as ensuring high quality, accurate recording to improve the quality of reports. These skills are equally necessary for other practitioners who come into contact with older people experiencing sexual abuse, particularly those in sexual violence practitioner roles.

There is a lack of existing research examining coping strategies of older people who experience abuse, particularly physical and sexual abuse. In this study, some practitioners expressed concern about the use of alcohol among older survivors, exacerbating existing physical and/or mental health problems, and creating additional support needs and challenges. Yet, a recent exploratory study of posttraumatic growth in a diverse sample of 1,863 adult female victims of sexual assault (Ullman, 2014) has shown that older age is associated with greater potential for posttraumatic growth, a concept that implies positive change following traumatic events. That individuals can grow following adversity is paralleled by evidence of gerotranscendance that is associated with increased life satisfaction in old age. However, T. Weiss (2014) argues that successful growth rests on avoiding a sense of rejection, receipt of social services, and having a sense of participation in society. There is a need for further research, which examines the coping strategies of older survivors.

Despite the acknowledged importance of interagency collaboration, none of the older people seen by practitioners in this study had been referred through age-related organizations (such as Age UK, The Silver Line, Action on Elder Abuse, or by adult safeguarding services). Referrals were from the police, health practitioners, or were self-referrals. This again reflects the emergence of elder abuse as a separate and distinct phenomenon from domestic and sexual violence, despite the obvious overlaps not only in definitions but in the perpetration and victimization of the abuse. In practice, this has led to an “ideological gulf” (Scott, McKie, Morton, Seddon, & Wassof, 2004, p. 7) meaning current support services are not working together, an issue acknowledged elsewhere (Mann et al., 2014). All 23 practitioners suggested that joint campaigns and joint training to raise awareness and encourage cross referrals between organizations may encourage older people affected by sexual violence to engage with services, echoing the findings in Mann et al.’s (2014) study. As Straka and Montminy (2006) previously argued, “a collaborative response is needed, accounting for both the age and gender dimensions of the problem” (p. 251). It is possible that useful lessons could be learned here from the work of the National Center for Elder Abuse (NCEA) in supporting the development of multiagency coalitions, networks, and task forces that bring together practitioners working across aging service providers, police, Adult Protective Services, elder law specialists, nurses, personnel from financial institutions, and domestic violence advocates. In the context of sexual violence, this provides opportunities for rape crisis centers, age-related organizations, the police, and adult protection services to work together to raise awareness of sexual violence in later life and develop clear referral pathways across and between these organizations to improve support provisions for older survivors.

A final implication arising from the study is the importance of recognizing that working with older people who have experienced sexual violence has an emotional impact on practitioners, something already acknowledged in relation to work with younger adult survivors of domestic and/or sexual violence (Baird & Jenkins, 2003; Dworkin et al., 2016; Schauben & Frazier, 1995; Slattery & Goodman, 2009) and children who have been sexually abused (Clemans, 2004; National Society for the Prevention of Cruelty to Children [NSPCC], 2013; Pack, 2004). Secondary or vicarious trauma (Baird & Jenkins, 2003; Schauben & Frazier, 1995) was described by five practitioners, who expressed the shock and distress they experienced working with older survivors and were uncertain about how best to support them. This was at least partly linked to the wider sociocultural attitudes toward older people and sex as well as “real rape” myths which practitioners had, to some extent, internalized themselves. This further reinforces the need for awareness raising of sexual violence against older people as well as tailored training and support for those who work with older survivors.

To conclude, this study builds on previous research by Mann et al. (2014) examining practitioner views and experiences of older sexual violence survivors. Although no generalizations can be made on the basis of interviews with the practitioners in this study, a number of the findings coalesce with evidence and arguments in recently published literature. This lends confidence that they can usefully serve to pose questions for further investigation. As Jordan (2013) has argued, our awareness and understanding of how individuals survive experiences of sexual abuse, experiences of criminal justice processes, and experiences of attempts to seek help are in their infancy. As a key variable in predicting resilience in older age (Blane, Wiggins, Montgomery, & Netuveli, 2011), the implications of diminishing supportive networks in older age, combined with wider social attitudes that serve to conceal and deny sexual violence against older people, should act as a clear signal to policy makers and researchers.

Practitioners’ perspectives of the effects of sexual violence on older people, and challenges in accessing and providing support, reinforce recent calls in the feminist literature to increase awareness of sexual violence and improve responsiveness as a way of moving toward prevention of sexual violence against older women (Fileborn, 2016). By adopting a critical feminist gerontological lens, a greater understanding of the ways age and gender intersect to create multiple challenges and effects for older survivors is developed; in this study, generational norms, values, and attitudes intersected with age-related health conditions and issues, and gender, to shape and influence the experiences, impacts, and challenges for older survivors and practitioners. A shift is also required in social and cultural attitudes to older age and gender that continue to stifle public discussion and awareness of sexual violence against older people, placing a very high price on personal disclosure (Mann et al., 2014).

There is an urgent need to develop more nuanced understandings of the effects of sexual violence, and how resilience can be promoted among older people who experience sexual violence. It is of critical importance that practitioners in the fields of social work and social care, health, criminal justice, specialist sexual violence and age-related organizations work together to develop knowledge of the circumstances and effects of sexual abuse for older people, and knowledge of how survivors can be supported to develop strategies for healthy recovery and long-term survival. Further research which examines the specific ways in which ageism and sexism intersect, utilizing feminist and gerontological frameworks, can develop our understandings of the range of impacts and support needs of older people and provide much-needed evidence to inform future policy and practice.

## Limitations

The data in this study were collected retrospectively from practitioners working in sexual violence organizations. The findings in relation to the impacts and effects of sexual violence for older survivors are based on practitioners’ observations and opinions and are limited to a relatively small number of cases, as people aged over 60 made up a small proportion of the overall number of survivors accessing their support services over the previous 5 years. Furthermore, although practitioners working with male survivors were included in this study, only two practitioners had this experience. Practitioners had very little experience of working with survivors from minority groups, including ethnic minority populations; therefore, the findings described in this article may not be reflective of those experienced across all older survivors, and there is a need for research which specifically explores the impacts and challenges for older survivors from minority groups.
